# Special Needs Dental Management of the Class 3 Obese Patient

**DOI:** 10.1155/2019/7976531

**Published:** 2019-02-10

**Authors:** Zanab Malik

**Affiliations:** Registrar Special Needs Dentistry, Department of Oral Medicine, Oral Pathology and Special Needs Dentistry, Level 3 Westmead Centre for Oral Health, Westmead, NSW, Australia

## Abstract

Obesity, classified as a chronic disease by the World Health Organisation (WHO), is a worldwide public health problem. Obesity has links with numerous systemic diseases which may complicate dental management and as such, patients with obesity and concomitant medical comorbidities are commonly managed by Special Needs Dentistry specialist departments in Australia. The sparsity of available evidence on the dental status in this group is likely due to significant access issues experienced by the class 3 obese, who often weigh >140 kg and therefore are unable to be examined or treated in conventional dental chairs. “Bariatric” is a term used to refer to a specific branch of medicine dealing with causes, prevention, and treatment of obesity. It is used widely in the literature to refer to obese patients; however, dentistry for this cohort (“bariatric dentistry”) is less well defined and represents less frequently used terminology. This case report is of a 58-year-old female, with class 3 obesity, who presented in May 2018 for outpatient consult to the Special Needs Unit/Medically Complex Dental Clinic at Westmead Centre for Oral Health, Sydney, Australia, with a compromised and neglected dentition and requiring full dental clearance. The case highlights many of the significant access issues and considerations for safe and effective delivery of dental management. As we move into the future, dental professionals need to become more aware of the growing challenge obesity presents and understand how medical complexities influence dental management. Facilities need to be able to meet this growing need and the specific requirements for a functional and safe bariatric dental service; dependent on both appropriate infrastructure and training.

## 1. Introduction

WHO defines obesity as “abnormal or excessive fat accumulation that may impair health” [1] and is commonly measured at the population level for adults using the body mass index (BMI). BMI is defined as weight in kilograms divided by the square of the height in metres. Although a clinically useful measure, BMI is limited in not accounting for differences in muscle mass, bone mass, and genetic makeup [2]. Obesity is determined by a BMI of greater than or equal to 30 kg/m^2^ with cut-off points adopted for use internationally by the WHO and based on associations between BMI, chronic disease, and mortality. The highest class of obesity, obese class 3, is defined as a BMI of equal to or greater than 40 kg/m^2^ with a very severe risk of comorbidities [1].

Limited current evidence recognises an association between obesity (not specific to class 3 obesity) and oral disease, namely, caries and periodontal disease, largely based on cross-sectional prevalence data [3, 4]. However, there are currently no evidence-based clinical guidelines for the dental management of patients with obesity. For those individuals who do not fall within the dental chair safe weight working limits, this necessitates the use of a modified dental chair and possible treatment planning modifications. These management considerations must therefore be extrapolated from case reports or series and expert opinion, identifying a need for further research in this area.

This case report will endeavour to provide further guidance for dental practitioners relating to the management (pre-, peri-, and post-operative) of patients with class 3 obesity. It will highlight the focus on comprehensive medical history taking and understanding the influence of medical comorbidities on dental treatment planning.

## 2. Patient Case

This is the case study of Mrs. D, a 58-year-old female who presented for outpatient consult to the Special Needs Unit/Medically Complex Dental Clinic at Westmead Centre for Oral Health, Sydney, Australia, referred by her general medical practitioner for specialist dental management. Her chief complaint was of multiple broken teeth and generalised discomfort from her teeth. She reported a history of right-sided facial swelling a few weeks prior, which had since settled with a self-administered course of antibiotics and increased dosage of her regular pain medications. The patient also reported her dentition was limiting her diet consistency to soft foods and significantly limited her social interactions. She expressed a desire for a “normal” appearance of her teeth and for denture rehabilitation in the future.

### 2.1. Comorbidities

Mrs. D's medical history was significant for class 3 obesity (BMI of 65 kg/m^2^). She was known to the local community obesity service since January 2018 which had resulted in successful weight loss of nearly 20 kg over the previous five months. She had metabolic syndrome and a plan was recommended by her endocrinologist for gastric banding surgery scheduled for November 2018. Mrs. D had moderate obstructive sleep apnoea (OSA) (confirmed via sleep study with no REM sleep in 2014, intolerant of CPAP machine) and reported considerable difficulty with sleep, requiring an upright posture. Remaining systemic history included borderline personality disorder (Abilify, Epilim), depression with a history of self-harm, and subsequent hospitalisation for suicide watch in 2005 (currently managed with Efexor, however, without psychology input). Mrs. D had well-controlled hypertension (Inderal, Frusemide), a history of iron deficiency anaemia (no current iron supplementation) and she experienced occasional migraines with aura, resulting in reduced vision/hearing and headaches (Aspirin prn on migraine onset).

Additionally, Mrs. D had subclinical hypothyroidism (Oroxine), gastro-oesophageal reflux disease (GORD) (Somac) with diverticular disease, urinary incontinence (managed with use of a bariatric commode), and chronic constipation (Coloxyl with Senna, Movicol prn). There was a history of non-alcoholic steatohepatitis (diet control) and renal impairment which was improving. She had osteoarthritis affecting both knees requiring total knee replacements; however, she was deemed unsuitable for surgical intervention due to her current weight (Targin, Panadol Osteo, Vitamin D). There were infected bilateral leg ulcers severely limiting Mrs. D's mobility, requiring use of a walker to mobilise. Her most recent hospitalisation was for emergency surgery following gall stone rupture in 2010. Mrs. D had no known drug allergies.

### 2.2. Social, Diet and Dental History

Mrs. D had no smoking history and did not imbibe any alcohol. She lived in Sydney in her family home with her husband and dog. She was engaged in employment as a tutor for secondary level mathematics. Her diet consisted of flavoured yoghurt and calorie-restricted meals/shakes. She had recently commenced drinking 600 ml of diet soft drink per day. Due to the nature of her dentition, Mrs. D had already transitioned to a soft diet. She had not engaged in oral hygiene for the last two years due to significant dental pain. Mrs. D had not had any dental intervention for many years as she was unaware of how to access a service with appropriate bariatric facilities and was embarrassed about her dentition. She subsequently avoided interaction with others and her only external engagement outside of her home for the last three years was for medical appointments.

### 2.3. Clinical Examination

**Figure 1:**
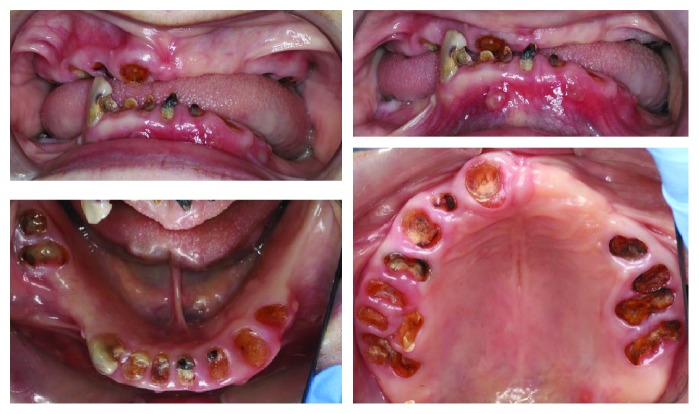
Intraoral photographs at time of consult. Buccal abscess adjacent to teeth 41 and 32 retained roots.

**Figure 2:**
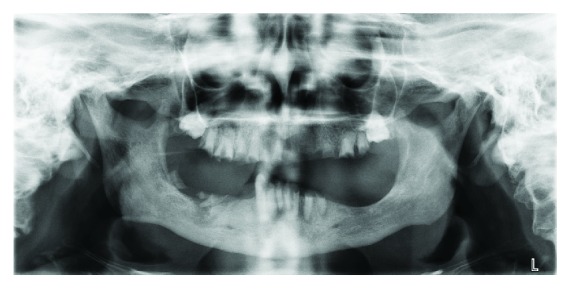
Orthopantomogram (OPG) July 2018 showing grossly carious retained roots and associated periapical pathology. Unerupted maxillary right and left third molars are also evident (18, 28).

Extra-oral examination at the time of consultation revealed no regional lymphadenopathy, trismus, facial swelling/asymmetry or TMJ pathology. A loss of occlusal vertical dimension was apparent. Baseline observations showed a blood pressure within normal range and oxygen saturation of 97% with Mrs. D seated upright. Clinical and radiographic intra-oral examination revealed generalised dry mucosa secondary to medication-related salivary gland hypofunction and buccal abscesses adjacent to mandibular right central and left lateral incisor retained roots (41, 32). Hard tissue examination revealed a grossly carious mandibular right canine tooth (43) with the remaining teeth evident as retained roots with hopeless prognosis (see Figure 1). Radiographic examination with OPG X-ray confirmed the clinical findings and showed evidence of unerupted maxillary right and left third molar teeth (18, 28) and multiple retained roots with associated periapical pathology (see Figure 2).

**Figure 3:**
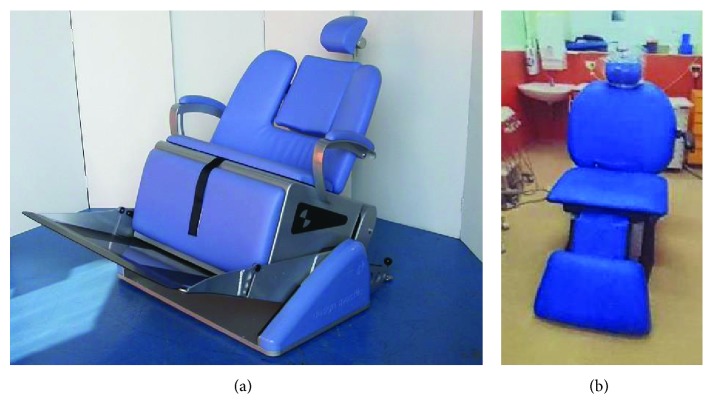
Differing styles of bariatric dental chairs, suitable for all patient groups up to 1000 lbs (454 kg) (image of “Barico” dental chair at Westmead Centre for Oral Health (a) and “bariatric bench” from Design Specific (b) https://www.designspecific.co.uk/html/b_bench_home.html).

Clinical examination and dental treatment was performed in a bariatric dental chair (see Figure 3).

### 2.4. Treatment Options

There was extensive discussion with Mrs. D regarding the available treatment options which included full dental clearance under local anaesthetic and construction of full maxillary and mandibular dentures following complete gingival healing post-operatively. We discussed realistic expectations surrounding prosthetic rehabilitation given no previous denture history for Mrs. D and reinforced continued soft diet in the interim. Mrs. D was agreeable to this treatment option.

The option of no treatment was discussed with ongoing risk of pain, swelling, and infection. Due to Mrs. D's high BMI and obstructive sleep apnoea, she presented a very high risk for management under sedation or general anaesthesia and a high risk of a medical emergency.

A preventive care plan was instituted by our oral health therapist, which included an antibacterial chlorhexidine gel and saliva substitutes (dry mouth gels) to reduce biofilm burden, stabilise dental disease risk, and assist in future denture use. Diet advice was also reinforced as part of healthy weight intervention.

### 2.5. Management of This Patient

Pre-operatively, blood tests were ordered to check coagulation studies and Mrs. D's bleeding risk on the background of hepatic dysfunction. They were within normal range to proceed with surgical dental intervention. A STOP-Bang score (scale to determine risk of OSA) of 6/8 confirmed Mrs. D's high risk of OSA consistent with her diagnosis. Mrs. D's baseline blood sugar level and blood pressure was also checked prior to commencing treatment. All appointment times for dental extractions were scheduled in the morning to allow for sufficient time to manage any potential complications.

Peri-operatively, mobilising Mrs. D into the facility, where the bariatric chair was located, required the use of a bariatric wheelchair. Mrs. D was reclined to a semi-supine position during treatment. Patient positioning was also assisted with cushions to help ease discomfort from lower limb pressure ulcers. Hoist facilities were readily available in case of a fall or medical emergency. Non-pharmacological behavioural management was used to alleviate anxiety and help reduce the risk of migraine. Registered nurse support was present for regular monitoring of vital signs especially pulse oximetry to monitor closely for desaturation. Full dental clearance (extraction of remaining 19 erupted teeth) under local anaesthesia over multiple visits was carried out uneventfully in chair. Mrs. D awaits full maxillary and mandibular denture construction at time of publication.

Post-operatively, it was imperative Mrs. D was discharged into the care of a responsible support person (her husband), who attended appointments with her. Appropriate analgesia therapy was recommended for post-operative pain management.

Although Mrs. D coped well with treatment under local anaesthesia, for many patients, given similarly extensive burden of treatment needs and presence of medical comorbidities, this may have necessitated dental treatment under intravenous sedation or general anaesthesia with specialist anaesthetist support, especially if in a setting of confounding dental phobia. In order to reduce anaesthetic risk, medical optimisation prior to surgery would be required. This could be via liaison with the specialist medical obesity service for pre-operative weight loss, the use of CPAP machine pre- and post-operatively on the background of OSA, and likely overnight admission and close monitoring for post-operative complications such as apnoea.

## 3. Discussion

This case report highlights several considerations in the dental management of patients with class 3 obesity like Mrs. D, who have many medical comorbidities that may require modifications to dental treatment planning.

With regards to cardiac and respiratory function, generalised obesity alters the total blood volume and cardiac function, whereas the distribution of fat around the thoracic cage and abdomen restricts respiratory function [4]. As such, sleep breathing disorders are common amongst obese individuals. OSA may be associated with thick, short necks and increased soft tissue presence surrounding the uvula [5]. A patent airway and satisfactory neck extension can be maintained via correct head positioning, as was ensured for Mrs. D, with regular monitoring of her oxygen saturation levels throughout treatment. However semi-supine positioning resulted in challenges for clinician and dental assistant positioning during aspects of treatment. It has been suggested to carry out a pre-operative assessment of OSA using a screening tool, such as the STOP-Bang score, prior to any treatment and especially prior to sedation or general anaesthetic for the obese patient [5]. Peri-operatively under general anaesthesia or conscious sedation, the presence of OSA complicates airway management and almost doubles the risk of the procedure for developing serious airway problems than the non-obese patient [5].

Other links with class 3 obesity include GORD due to the impaired communication between the lower oesophageal sphincter and the stomach [2]. There is a dose-dependent association between increasing BMI and GORD [6] and may present a risk of aspiration of gastric contents if treatment is performed under general anaesthesia. Mrs. D had metabolic syndrome increasing her risk of diabetes and cardiovascular disease. There is evidence for intra-abdominal fat deposits as a major contributor to the development of hypertension, elevated plasma insulin concentrations, insulin resistance, diabetes mellitus, and hyperlipidaemia [2, 7]. Ensuring satisfactory blood pressure and blood sugar levels prior to commencing invasive dental treatment such as dental extractions is crucial. Further control may be facilitated through diet and weight management, and the dental team is in an ideal position to provide preventive advice [8].

Excess weight and load on joints predispose patients with obesity to osteoarthritis [9]. The literature suggests that chronic pain and obesity are linked comorbidities [10], adversely impacting each other [9]. Obesity appears to be a risk factor for developing various pain diagnoses including headaches, lower back pain, fibromyalgia, abdominal pain, pelvic pain, and neuropathic pain [9]. Weight loss for obese pain patients appears to be an important aspect of overall pain rehabilitation, although more research is needed to determine strategies to maintain long-term benefits [9].

The association between obesity and mental illness is complicated and bi-directional. Obesity is linked to an increased risk of a psychiatric diagnosis, and in turn, mental illness (or its pharmacological management) may precipitate and perpetuate weight gain and obesity [11]. Some evidence has indicated that the relationship between weight and mental illness is dose dependent [11]. Further compounding Mrs. D's caries risk, many antidepressant and antipsychotic medications have anticholinergic properties which likely contributed to the evident salivary gland hypofunction.

In addition, obesity is unfortunately often associated with negative social consequences. Weight-related stigma, both by others and self-imposed, and internalized anti-obesity attitudes have been found to be associated with emotional distress including symptoms of anxiety and depression [12]. Mrs. D voiced extreme anxiety regarding access and attendance for dental services and her oral health became neglected as a result.

Similarly, one can hypothesise that there are higher levels of oral health problems in the class 3 obese population. However, data supporting this hypothesis is currently lacking. As a result, there may be considerable impact on quality of life [13] which can be further compounded by the loss of teeth [14]. Qualitative research in the emotional effects of complete tooth loss has revealed a wide range of reactions such as bereavement, loss of self-confidence, concerns about appearance and self-image, and a lack of preparation for the loss of teeth [14]. For Mrs. D, this needed to be considered in the context of her long-standing history of anxiety and depression. A post-operative support network and pain management plan is required to minimise these effects, as in the case of Mrs. D. It has been shown consistently that obesity can negatively impact important aspects of health-related quality of life with higher degrees of obesity associated with greater impairment [12]. As demonstrated in the case of Mrs. D, there is a suggested implication that quality of life related to oral health may also deteriorate [15], although this has never been quantitatively measured in a population with obesity.

## 4. Conclusion

Dental professionals need to develop an understanding of the growing challenges that patients, particularly those with class 3 obesity, present for comprehensive dental management, taking into account their medical complexities. Patients should be referred in a timely manner to appropriate facilities where bariatric dental chairs are available. Patients and their corresponding specialist medical obesity services should be educated about available facilities and funding increased to ensure growing demand can be met. Multidisciplinary holistic management for these patients may necessitate specialist Special Needs Dentistry input for the safe delivery of dental treatment. The paucity of literature related to obesity and dental disease highlights a need for further research in this population.
